# The development of academic family medicine in central and eastern Europe since 1990

**DOI:** 10.1186/1471-2296-14-37

**Published:** 2013-03-19

**Authors:** Anna Krztoń-Królewiecka, Igor Švab, Marek Oleszczyk, Bohumil Seifert, W Henry Smithson, Adam Windak

**Affiliations:** 1 Department of Family Medicine, Chair of Internal Medicine and Gerontology, Jagiellonian University Medical College, Krakow, Poland; 2 Department of Family Medicine, Faculty of Medicine, University of Ljubljana, Ljubljana, Slovenia; 3 Department of General Practice, First Medical Faculty, Charles University in Prague, Prague, Czech Republic; 4 Academic Unit of Primary Medical Care Medical School, University of Sheffield, Sheffield, UK

**Keywords:** Family medicine, General practice, Medical education, Medical research, Central and eastern Europe

## Abstract

**Background:**

Since the early 1990s former communist countries have been reforming their health care systems, emphasizing the key role of primary care and recognizing family medicine as a specialty and an academic discipline. This study assesses the level of academic development of the discipline characterised by education and research in central and eastern European (CEE) countries.

**Methods:**

A key informants study, using a questionnaire developed on the basis of a systematic literature review and panel discussions, conducted in 11 central and eastern European countries and Russia.

**Results:**

Family medicine in CEE countries is now formally recognized as a medical specialty and successfully introduced into medical training at undergraduate and postgraduate levels. Almost all universities have FM/GP departments, but only a few of them are led by general practitioners. The specialist training programmes in all countries except Russia fulfil the recommendations of the European Parliament. Structured support for research in FM/GP is not always available. However specific scientific organisations function in almost all countries except Russia. Scientific conferences are regularly organised in all the countries, but peer-reviewed journals are published in only half of them.

**Conclusions:**

Family medicine has a relatively strong position in medical education in central and eastern Europe, but research in family practice is less developed. Although the position of the discipline at the universities is not very strong, most of the CEE countries can serve as an example of successful academic development for countries southern Europe, where family medicine is still not fully recognised.

## Background

Before the collapse of communism in Europe at the end of the nineteen eighties health care systems in most of central and eastern European (CEE) countries were very similar. They followed to a greater or lesser degree the Soviet-style centralized Semashko model, which was dominated by specialists and hospital services, and ill-prepared to provide effective health care to individuals and populations [1]. The Semashko influence was profound in countries that were previously an integral part of the old Soviet Union while countries of former Yugoslavia had a different system, based on ideas of Andrija Štampar [2-4]. In the last 30 years all former communist countries have reformed health systems and have emphasized the key role of primary care and recognized family medicine as a specialty and an academic discipline [5,6]. Post-communist countries in Europe had many common experiences in the process of health care reform, but not surprisingly given the pre-reform variation, the development of general practice/family medicine in different areas was not identical [2]. This mirrors variation in primary care in Western Europe since the Second World War with some countries such as the UK and Spain embracing the concept of a state or national health system (the Beveridge model) while others following the insurance (Bismarck) model with less state influence [7]. The position of family medicine varies between and within systems but is dependent on the gate-keeper role [8] and strong professional/scientific representation.

The academic position of family medicine is an important element when assessing the position of family medicine in the CEE countries [6]. Education is identified as one of the features of workforce development, which then determines the structure of primary care [9]. Teaching and research are important academic activities in developing family medicine/general practice in this region of Europe but have not yet been reviewed and compared. There are few papers describing academic family medicine in the region or other fields of primary care [6,10] and few limited to a particular country [11,12]. This is in contrast to Western Europe where family medicine is perceived as a relatively well-established academic discipline [13,14] that provides proven health outcome benefits in countries with strong primary care [15].

The aim of this study is to assess and compare the development of teaching and research in general practice/family medicine in post-communist Europe. The study aims to answer the following questions:

1. What is the variation between different CEE countries in the development of family medicine in undergraduate and postgraduate medical education and research? 2. To what extent is the academic profile of general practice developed and recognized in this part of Europe?

## Methods

### Design

The study was designed as a cross sectional key informants survey to be conducted in 14 post-communist European countries. Key informants were identified and provided data from Bulgaria, Croatia, Czech Republic, Estonia, Hungary, Lithuania, Montenegro, Poland, Romania, Russia, Slovakia and Slovenia. Data collection was not forthcoming from key informants in Latvia and Serbia. The aim is to explore the state of development of family medicine/general practice (FM/GP) in the following areas: (1) role in healthcare system, (2) quality of care, (3) medical education and (4) research. The data provided by the respondents were validated against other sources of information including medical literature, websites, and official documents available nationally or published internationally.

The study was conducted within the framework of the international research project ‘Family Medicine After Transformation in Middle and Eastern Europe’ (FATMEE), partially founded by the scientific grant provided by the European branch of the World Organization of National Colleges, Academies and Academic Associations of General Practitioners/Family Physicians (WONCA Europe). Because of the non-experimental design of the study, and based on the opinion on ethical issues from the lead institution (the Jagiellonian University Krakow) further approval from Ethical Committee had not to be sought.

This paper is one of a pair of papers and reports the development of medical education and research in CEE countries, while the results of the other part of the study illustrating the role of FM/GP in healthcare system and quality of care is published elsewhere [16].

### Respondents

In each country a pair of key informants was recruited. Every participant is a high-level expert, well-acquainted with the national primary healthcare in their country. The national key informants were recruited independently from different academic centres and their identity was not revealed one to another. The key informants were selected by approaching representatives of national scientific organizations belonging to WONCA Europe to identify the best candidates. Additionally in some countries representatives of WONCA Europe network organizations (EURACT, EQuiP, EGPRN) were involved in this process.

### Instrument

**Figure 1:**
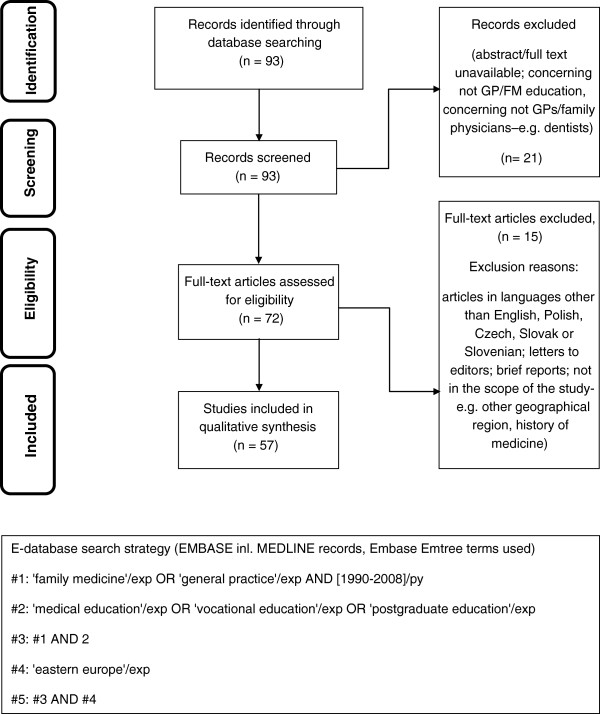
Systematic literature review flow diagram in area of Education and E-database search strategy.

**Figure 2:**
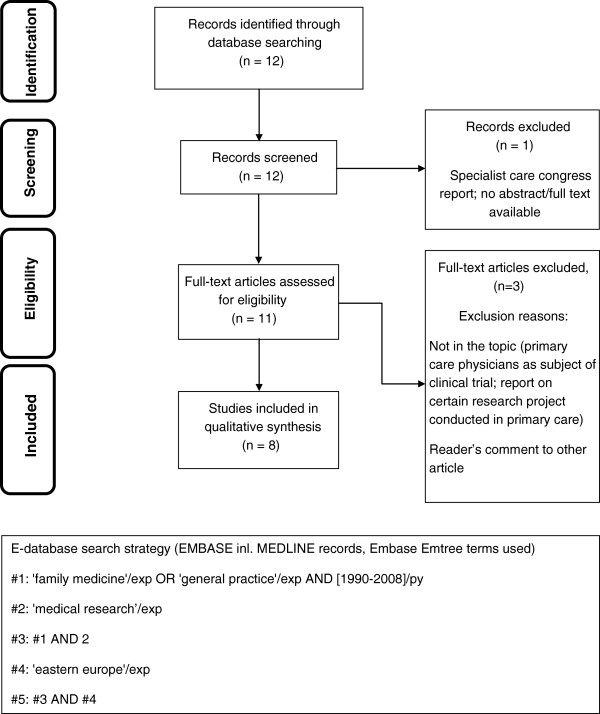
Systematic literature review flow diagram in area of Research and E-database search strategy.

A systematic literature review was performed as an initial step to identify themes to be incorporated into the questionnaire. E-databases (Pub-Med, Embase) were searched using a structured search strategy, focusing on geographical criteria and the specific Emtree terms and their combinations. Details of the e-databases search were previously presented [16]. Based on the initial search results and screening procedure 93 papers in the Education and 12 in the Research area respectively were included for qualitative assessment. Two researchers working independently assessed eligibility of the papers and finally achieved consensus. 57 relating to medical education and 8 relating to research were considered eligible for the initial development of the study instrument. The literature review process is presented on flow diagrams (adapted from PRISMA statement [17]) in Figures 1 and 2.

Based on the literature review and a series of panel discussions to confirm themes the research team achieved consensus and drafted an initial preliminary version of the questionnaire, exploring the four FATMEE areas of FM/GP development. The medical education area consisted of 18 major indicators retrieved from 57 papers, measured by 22 questions, related to teaching and learning at all levels – from basic medical education (BME) through vocational training (VT) to continuing professional development (CPD). The research section included 7 major indicators retrieved from 8 articles related to development of family medicine as a scientific discipline measured by 11 questions. The teaching and research part of the questionnaire was composed of 15 open-ended questions and 18 close-ended questions, among which 9 were simple “yes” or “no” questions and 9 in multiple choice format. There was a special comment field to each question, for free text Additional file 1. The draft version of the questionnaire was piloted in 3 countries of the region (Poland, Czech Republic, Slovenia) to check consistency and face validity. The final version was developed after thorough consideration of all comments and remarks.. A web based version of the questionnaire was constructed using a commercial version of the Survey Monkey® online tool.

### Data collection and analysis

The first round of data collection was conducted between June and August 2010. The key informants were asked to complete the web-based questionnaires, and the results from the first round were downloaded and analysed. Responses of both informants from each country were compared. Questions with missing, conflicting or opposing answers were selected for the second round of data collection. The questions were returned to both national respondents, who were asked to review the problematic issue and compare their own response with the anonymous counterpart’s answer. The respondents were encouraged to provide any known sources of information to support their answers. Whenever possible these discrepancies were presented to the partner informant for further consideration. The second round of data collection was conducted between November 2010 and March 2011. Only fully agreed issues were included in the final analysis.

## Results

### Profile of the respondents

In total responses from 24 informants were included in the analysis. The majority (83%) were physicians, except in Bulgaria, Czech Republic, Lithuania and Russia, where one of the experts was another health care professional. The mean age of the respondents was 50 years (range 42-64 years), 13 of them were men. The scientific experience of the informants varied. Except for one respondent from Montenegro, all of the respondents had a scientific degree: one third of them were awarded Medical Doctor (MD) or Master of Science (MSc) title, 3 respondents had Philosophy Doctor (PhD) title, every fourth informant was a professor, 5 of them were post-PhD/associate professors, and one respondent from Romania did not specify a scientific degree. 20 experts from 11 countries declared family medicine as their first medical specialty. Six of them had additionally a second medical specialty. Eighty four per cent of the respondents worked in primary care, half of them combined the work in primary care with work at university medical school, and 16% were employed only by a university.

### Inter informant agreement

It was judged that the information from an expert in each country is correct only if the counterpart’s answer was the same. After two rounds of data collection there was disagreement in some questions. Opposite or missing issues were not included in the final analysis. Table 1 shows the percentages of agreed questions of teaching and research section of the questionnaire in each country. The mean percentages of agreed questions in both sections were 82%, on average there were 18/22 agreed teaching questions and 9/11 in the research questions.

**Table 1:** Inter-experts agreement by country and study’s area

**Country**	**Questions agreed**	
	**Education** **N (%)**	**Research** **N (%)**
Bulgaria	21 (95%)	11 (100%)
Croatia	12 (55%)	5 (45%)
Czech Republic	21 (95%)	8 (73%)
Estonia	21 (95%)	9 (82%)
Hungary	19 (86%)	10 (91%)
Lithuania	20 (91%)	8 (73%)
Montenegro	18 (82%)	10 (91%)
Poland	14 (64%)	7 (64%)
Romania	20 (91%)	10 (91%)
Russia	10 (45%)	9 (82%)
Slovakia	22 (100%)	11 (100%)
Slovenia	18 (82%)	6 (55%)

### Education

#### Teaching infrastructure

**Figure 3:**
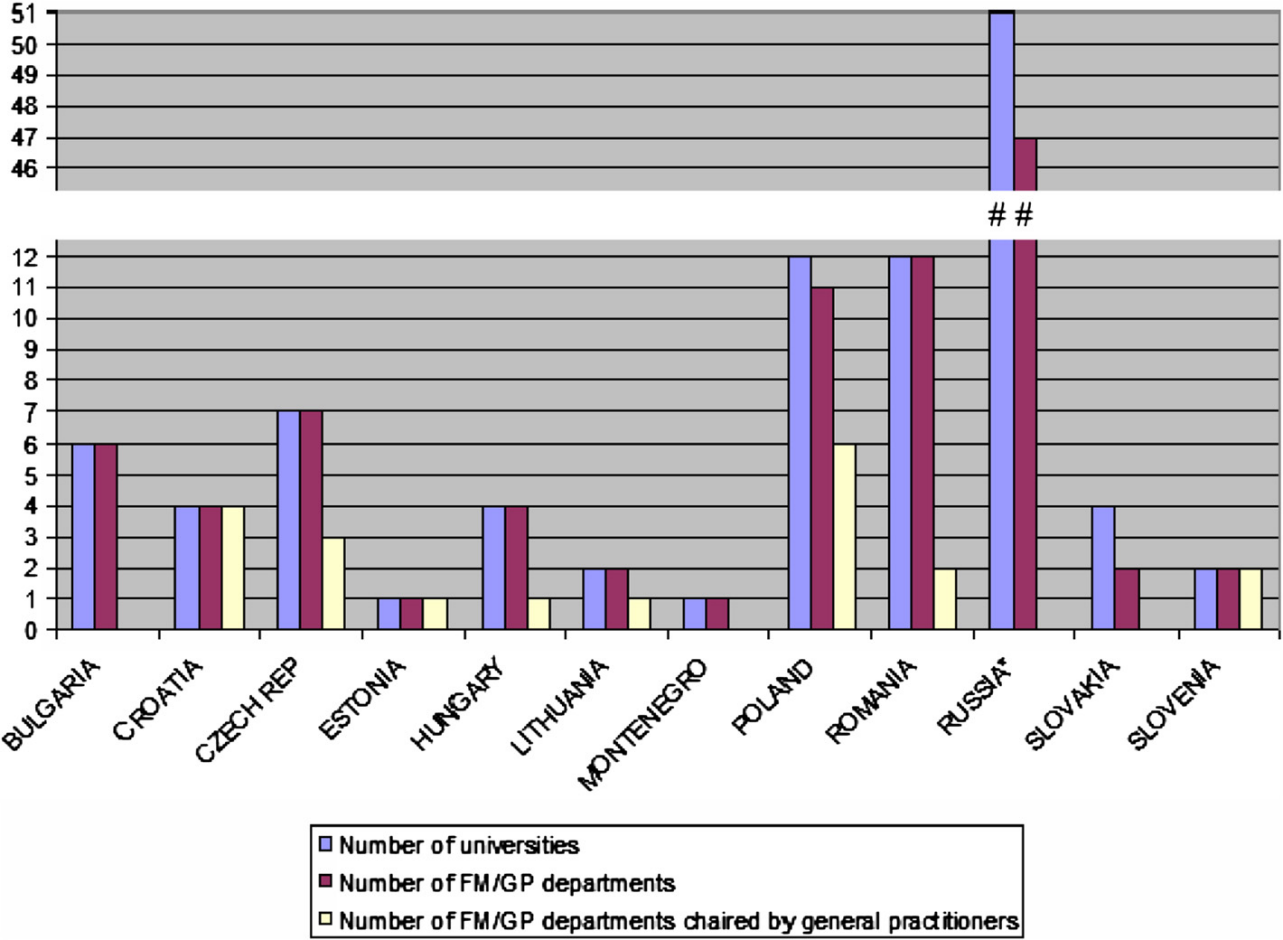
Overview of FM/GP position in universities.

The number of family medicine departments equals the number of universities in all of the countries except Poland, Russia and Slovakia. The FM/GP department was chaired by a general practitioner in only Croatia, Estonia, Lithuania and Slovenia. Professors of other medical disciplines lead 6 of 11 departments in Poland and the majority of the departments in Czech Republic, Hungary and Romania. In Bulgaria, Montenegro and Slovakia none of the FM/GP departments is led by a family physician. All university departments are responsible for basic medical education. The specialist training in the field of FM/GP is conducted in all medical faculties at university level in Bulgaria, Estonia, Hungary, Lithuania and Poland, in 50% of universities in Croatia and 25% in Slovakia. The overview of the position of family medicine in universities is presented in, Figure 3.

There are some quality measures and requirements to be fulfilled in teaching practices in Bulgaria, Czech Republic, Estonia, Hungary, Montenegro and Slovenia. In Slovakia, similar requirements have been developed but not yet applied. Accreditation to teach is necessary in 4 countries, (Bulgaria, Czech Republic, Estonia and Hungary). The information about all other countries was inconsistent.

In all the countries, except Slovakia, there are some courses for FM/GP teachers available. Most of them are organised infrequently, mainly as results of international projects. No long term sustainability of the courses is currently secured in any of the studied countries.

#### Undergraduate education

Family Medicine/General Practice is part of the undergraduate medical curriculum in all participating CEE countries. In all but 2 of the studied countries every university offers an undergraduate program in FM/GP. The exceptions are the Czech Republic, where 3 of 7 universities teach FM/GP and Russia, where FM/GP is a subject in only 13 of 51 medical faculties. In a minority of the countries the minimum number of teaching hours in FM/GP is guaranteed by regulation. In the medical curriculum in Estonia there is a fixed number of teaching hours in family medicine. Table 2 shows more detailed data about undergraduate programmes.

**Table 2:** Academic structures and time limit for undergraduate teaching in the field of FM/GP

**Country**	**No. of universities**	**No. of universities with fm/gp undergraduate programme**	**No. of teaching hours in fm/gp**
Bulgaria	6	6	Min. 60
Croatia	4	4	Min. 180
Czech Republic	7	3	No regulation
Estonia	1	1	80
Hungary	4	4	80-100
Lithuania	2	2	n.a.
Montenegro	1	1	60
Poland	12	12	Min. 105
Romania	12	12	No regulation
Russia	51	13	108*
Slovakia	4	4	No regulation
Slovenia	2	2	Min. 200

n.a.- data not available.

* to whole primary care, not GP/FM exclusively.

#### Specialist training

Specialist family medicine training is organised by universities in all countries, except Slovenia, where a Medical Chamber is in charge of specialist training and the Czech Republic, where there is a special postgraduate centre. In Montenegro a postgraduate programme formally exists, but did not start before data collection was completed. Special postgraduate centres organise specialist training in FM/GP on an equal basis with universities in Poland, Romania, Russia and Slovakia. In Poland and Slovakia hospitals are also authorized as teaching institutions for future general practitioners. Certain central institutions are partially involved in the specialist training’s organization in Hungary (National Institute of Primary Care) and in Romania (The Directorates of Public Health). An overview of the institutions responsible for organisation of specialist training is presented in Table 3.

**Table 3:** Providers of specialist training in family medicine/general practice

**Country**	**Special postgraduate centres**	**Universities**	**Hospitals**	**Other**
Bulgaria		x		
Croatia		x		
Czech Republic	x			
Estonia		x		
Hungary		x		National institute of primary care
Lithuania		x		
Montenegro		x		
Poland	x	x	x	
Romania	x	x		The directorates of public health
Russia	x	x		
Slovakia	x	x	x	Teaching practices
Slovenia				Medical chamber

**Figure 4:**
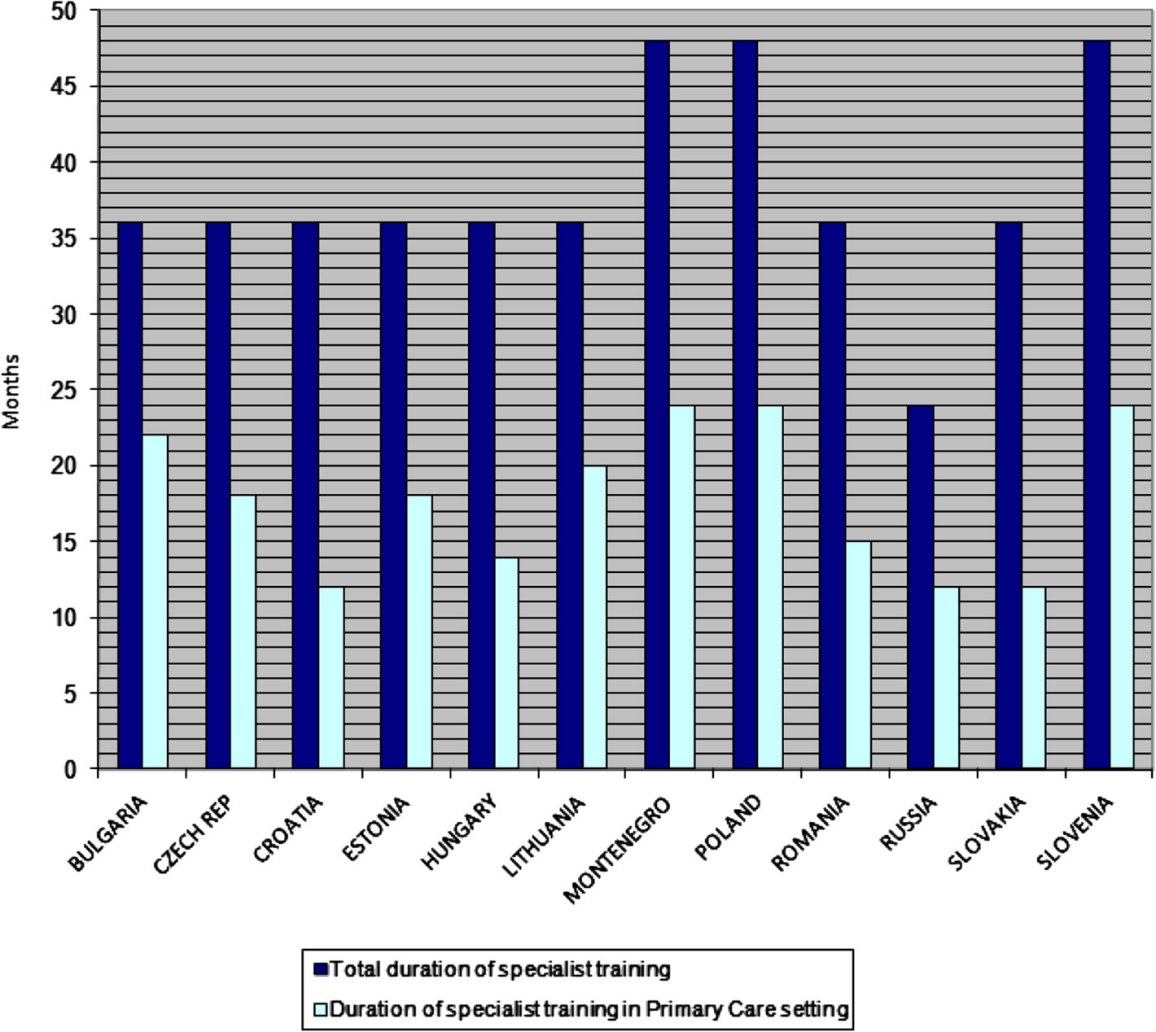
Duration of specialist training in family medicine/general practice.

The length of the training programme for general practitioners is 3 years in most countries, apart from a 4 year programme in Poland, Slovenia and Montenegro, and a 2 year programme in Russia. Future general practitioners spend half of their specialist training time in a Primary Care setting in Czech Republic, Estonia, Montenegro, Poland, Russia and Slovenia. In the other countries the duration of specialist training in a Primary Care setting is shorter (Figure 4).

Motivations to start vocational training in FM/GP differ between the countries. In Bulgaria, Czech Republic, Estonia, Hungary, Lithuania, Poland, Romania and Slovakia there is easy access to specialization in family medicine. For alumni from Bulgaria, Czech Republic, Estonia, Hungary, Lithuania and Montenegro the important driver is a better perspective for professional development than in other medical specialties. In the Czech Republic FM/GP trainees and general practitioners receive a higher salary than some other specialties, and specialization in this field is a fast track to private practice. In Estonia doctors are allowed to work as independent contractors but only those who are accredited as a specialist in family medicine can have a patient list. There are no special incentives to undertake specialty training in FM/GP in Russia.

Trainees in family medicine are paid by state or local government in the majority of countries (Table 4), except Slovenia, where they are paid by an insurance company and Slovakia, where residents are financed by trainers and hospitals. In Bulgaria the state budget pays universities, hospitals and primary care settings for their expenses and the education is free for the trainees, but they are not paid a salary.

**Table 4:** Payers of residents’ salary during vocational training in family medicine/general practice

**Country**	**No payment**	**Trainers**	**State or local government**	**Insurance companies**	**Hospitals**
Bulgaria	x				
Czech Republic			x		
Estonia			x		
Hungary			x		
Lithuania			x		
Montenegro			x	x	
Poland			x		
Romania			x		
Russia			x		
Slovakia		x			x
Slovenia				x	

In all countries the specialist training ends with a formal exam in various formats. In Bulgaria, Croatia, Czech Republic, Montenegro, Poland and Slovenia the knowledge of the trainees is tested with multiple choice questions (MCQ). In contrast, in Estonia and Romania there are written exams. Both forms of the theoretical exam (MCQ and written exam) are used in Hungary (planned), Lithuania, Russia and Slovakia. In Estonia the assessment also involves video-analysis and scientific project. There is a practical exam in every country apart from Poland and an oral exam in all countries except Bulgaria. In some departments in Russia objective structured clinical examination (OSCE) is used.

#### Continuing professional development

There is a formal re-certification procedure of Family Physicians/General Practitioners in Croatia, Estonia, Hungary, Lithuania, Poland, Romania, Russia, Slovakia and Slovenia. It is mostly based on credits awarded for participation in various educational activities. In most of the countries it is obligatory, but is voluntary in Estonia and Poland. Re-certification procedures do not exist in Bulgaria, Czech Republic and Montenegro.

### Research

Postgraduate research programmes (Ph.D.) in FM/GP are available in Lithuania and Poland, where 5 family physicians or more take part each year. Programmes also function in Bulgaria and Slovakia, but with less than 5 general practitioners yearly in the program. In Estonia family physicians participate in the PhD programme at the Medical Faculty of the Tartu University. It is a common programme for all specialties and the research topics are specific for the discipline. General practitioners from the Czech Republic, Montenegro, Romania and Russia do not have their own PhD- programmes.

Scientific organisations of Family Physicians/General Practitioners exist in all countries except Russia. The scientific organisation in Montenegro has yet to be operational.

Scientific peer-reviewed journals on FM/GP are published in Hungary- 12 issues per year, in the Czech Republic and Lithuania- 10 issues per year, in Bulgaria- 6 issues per year, and 4 issues a year in Poland and Russia.

National scientific conferences are regularly organised in all the countries. In the Czech Republic, Poland, Romania, Russia and Slovenia they are organised at least twice a year. In Croatia, Estonia, Hungary and Slovakia a scientific conference takes place annually, and in Bulgaria national congresses are organized every second year and smaller regional conferences annually. In Montenegro there are no national conferences.

Only the Czech Republic has special funds to carry on research in FM/GP. None of the other countries offers research grants exclusively reserved for the studies in FM/GP.

Research networks of Family Physicians/General Practitioners function in Bulgaria, Czech Republic, Croatia, Hungary and Poland. In other studied countries organized research networks of practices do not exist.

## Discussion

### Summary of main findings

During the last two decades family medicine in central and eastern Europe has been formally recognized as a medical specialty and successfully introduced into the medical curriculum. All countries have identified the need for teaching family medicine and recognized general practice as an essential element of undergraduate medical education. Family medicine is now a part of the medical curriculum in each studied CEE country, but only a few have stipulated the minimum number of teaching hours in FM/GP. Family Medicine/General Practice teachers participate in some teaching courses, which are organized within the scope of international projects; nevertheless no sustainability of the courses is secured in any of the studied countries. As far as medical specialization in family medicine is concerned, there are vocational training programmes organised in all the countries, with a formal end point examination. The minimum duration of specialist training in central and eastern Europe, apart from Russia, fulfils the recommendations of the European Parliament [18]. Teaching family medicine is progressively developing but research activity is relatively weak. Research activity in the field of general practice has started recently, but the lack of an academic tradition in this field and shortage of relevant infrastructure, do not encourage research careers for general practitioners. Academic departments are often chaired by specialists of other disciplines, holding scientific titles or degrees, with little or no background in primary care. Scientific organisations of Family Physicians/General Practitioners function in almost all countries except Russia. National scientific conferences are regularly organised in all countries. Scientific peer-reviewed journals on FM/GP are published only in a half of the studied countries. None of the countries in the region, apart the Czech Republic, offers special research grants for studies in FM/GP.

### Strengths and limitations of the study

The study was performed as a cross sectional key informants survey, developed in line with this kind of survey methodology [19]. One of the main advantages of this methodology is that good quality data can be collected in a relatively short period of time [20] with limited resources. It also allows access to grey literature and documents published only in national languages, normally unavailable for international research [19]. Careful selection of high level experts and the necessity to gain mutual agreement also increased the chance of gathering reliable data, additionally validated by the information collected within the systematic literature review. An advantage of this method is the relatively low cost for extensive data collection that is suitable for international comparisons [21,22]. On the other hand the reliability of data would improve if there are resources (time and money) to invite more in-country experts. The disagreement between two informants left us with white spots in some areas. The failure of recruitment of key-informants in other than presented CEE countries make the picture somehow incomplete and unfinished. That is the challenge for future investigations. Although the researchers at the initial stage took all necessary measures to develop the adequate questionnaire, it is acknowledged that the tool was not fully validated. We think that this is the largest study yet presenting academic development of FM/GP in central and eastern Europe.

### Contextualisation of key findings

Health reforms in the former communist countries of central and eastern Europe have introduced a Western European model of primary care delivered by general practitioners [5,23,24]. The General Medical Council’s publication ‘Tomorrow’s Doctors’ emphasized the need for undergraduate medical education in general practice for the first time in the early 1990s [25]. This guidance influenced the medical curriculum content not only in United Kingdom, but also in other countries and led to the recognition of general practice as an essential element of undergraduate medical education in Western Europe. Such a policy is now strongly recommended for the whole of Europe [26]. In central and eastern Europe family medicine is now a part of medical curriculum in each country, but in comparison to the United Kingdom, the Netherlands and Nordic countries and the academic position of family medicine is not yet well established. In the United Kingdom each medical school has a FM/GP department -all with at least one professor of family medicine [27]. Our findings shows that in CEE countries almost all universities have FM/GP departments, but only a few of them are chaired by general practitioners and this situation is unfortunately likely to remain stable [6,10]. Recognition of family medicine as an academic discipline in the United States has a history of almost 40 years and is now coming to a new phase in order to adapt better to new challenges and expectations of patients in the 21^st^ century [28]. The countries of northern and western Europe, with strong primary care systems, have a long tradition of general practice research, while this area is still not well developed in southern part of Europe [29-31]. A crucial role in the scientific development of FM/GP is in the development of practice-based research networks [32]. Their creation is still a challenge for most of the CEE countries. When they do not exist, it is important that networks in primary care are developed, based on projects of common interest. This is an important role for international organisation of family medicine such as the European Primary Care Research Network (EGPRN) [33-35].

There are several regions in Europe, where family medicine has not yet been fully recognized as an independent academic discipline. This issue concerns particularly Mediterranean countries, where there is poor financial and academic support for the development of family medicine [36]. Most of the CEE countries have reached a much higher level of academic development of the discipline and could share their experiences in the process of change in Southern Europe, especially in the area of teaching [37].

## Conclusions

The teaching of family medicine in CEE countries has a relatively well-established position, while the academic and research profile develops slowly.

The results of this study confirm awareness of the growing role of general practice in medical education. Most of the CEE countries, especially those which have joined the European Union, can serve as an example of successful development in this field for southern European countries, where family medicine is still not fully recognised. However, academic family medicine in post-communist Europe is still underdeveloped in comparison with northern and western European countries, where more effective health care systems rely on a pivotal role of family medicine.

Further academic development of the discipline, crucial for the future of family medicine in CEE countries, requires a greater focus on research. Policy decisions and investment are needed to attract young physicians not only to clinical practice in family medicine, but also to a future university career.

## Competing interests

The authors declare no conflict of interest.

## Authors’ contributions

AKK has been involved in data analysis, drafting and revision of the manuscript. IS was involved in conceptual work, study design, data collection, drafting and revising of the manuscript. MO contributed in conceptual work, study design, data collection, data analysis, drafting and revising of the manuscript. BS has contributed in conceptual work, study design, data collection, drafting and revising of the manuscript. WHS has contributed in drafting and revising of the manuscript. AW had a substantial contribution in conception and design of the study, data collection and analysis, drafting and revising of the manuscript. All co-authors have given the final approval of the version to be published.

## Pre-publication history

The pre-publication history for this paper can be accessed here:

http://www.biomedcentral.com/1471-2296/14/37/prepub

## Supplementary Material

Additional file 1 The family medicine in Middle and Eastern Europe (FATMEE) study instrument. Click here for file
