# Supplementary material for: The development of academic family medicine in central and eastern Europe since 1990
Source: BMC Fam Pract. 2013 Mar 19;14:37. doi: 10.1186/1471-2296-14-37 (PMC3608318; doi:10.1186/1471-2296-14-37)
Supplement: Additional file 1 — The family medicine in Middle and Eastern Europe (FATMEE) study instrument. [file 1471-2296-14-37-S1.pdf]

The Family Medicine in Middle and Eastern Europe (FATMEE) Study instrument.

No.	Indicator		Comments
**Area I. Role in Health Care System**			
**1.1**	Is there an official policy document presenting current role of family medicine in your country? If yes, what kind of document is it?	[parliamentary law/ministerial decree/ scientific paper/other]	
**1.2**	Is there an official policy document presenting future development of family medicine in your country? If yes, what kind of document is it?	[parliamentary law/ministerial decree/ scientific paper/other]	
**1.3**	Are the competences of family physicians clearly described? If so, on which level?	[Government/insurance company/college/local authorities/other]	
**1.4**	Who is responsible for organization of family medicine/general practice services?	[government/local authorities/insurance companies/other - name]	
**1.5**	Is specialty in family medicine/general practice formally recognized?	[Yes/No]	
**1.6**	If so, has it the same formal status as other specialties (e.g. neurology or cardiology)	[Yes/No]	
**1.7**	Do formal requirements exist for physicians (such as GPs/Family doctors) to work in PC?	[Yes/No]	
**1.8**	What kind of physicians can provide PC services?	[family physicians/ internists/ pediatricians/ gynecologists/ other specialists/ physicians without any specialization]	
**1.9**	What percent of all PC physicians have specialization in family medicine?		
**1.10**	What is the formal status of family physicians	[only state employee/mostly state employee/both state employee and independent contractor/mostly independent contractor/only independent contractor]	

**1.11**	Total expenditure on PC as % of total expenditure on health			
**1.12**	Number of GP consultations per capita per year			
**1.13**	1.13.1	Do PC physicians have their personal patient lists?	[yes/no]	
	1.13.2	If [yes], what is an average list size?	[*n*]	
	1.13.3	Is there a minimum and maximum limit?	[yes: (n_{min}); (n_{max})/ no]	
	1.13.4	Are there any measures (e.g. in payment) employed to influence the maximum or minimum size of the list?	[yes/no]	
	1.13.5	If [yes] describe briefly		
**1.14**	1.14.1	How PC services are paid?	[fixed budget/pure capitation/capitation with wages/fee for service/mixed system]	
	1.14.2	If mixed system, what are the components?		
**1.15**	1.15.1	How family physicians are paid?	[salary/ income from the contract/ by patients out of pocket/ mixed system]	
	1.15.2	If mixed system, what are the components?	[*please describe briefly*]	
**1.16**	Are family physicians gate-keepers to specialist services?		[total/partial/ not at all]	
**1.17**	Which of the following specialties are accessible without referral from family physicians?		Gynaecologist /obstetrician<br>Paediatrician<br>Internist<br>Ophthalmologist<br>ENT specialist<br>Oncologist<br>Dermatologist<br>Surgeon<br>Dentist<br>Psychiatrist<br>Other (name)	

**1.18**	1.18.1 Is there a number weekly of working hours/doctor's availability in practice regulated?	[yes/no]	
	1.18.2 If [yes], how much is it?	[*n*]	
	1.18.3. If [yes] are doctors obliged to spend number of hours in office, how many?	[yes/no] 1.18.4 [*n*]	
**1.19**	Is out- of- hour care (nights, weekends, holidays & festivals) part of PC contract?	[obligatory/voluntary/ not at all]	
**1.20**	How is out-of-hour care organized:	1. Rota of duties shared by several practices (physician)<br>2. Deputizing service hired<br>3. Emergency service<br>4. Hospital-based centres<br>5. Other *(please describe briefly)*	
**1.21**	What kind of services are normally provided by family practices?		
	1.21.1 Curative care for children	[always/ sometimes/ never]	
	1.21.2 Curative care for adults	[always/ sometimes/ never]	
	1.21.3 Minor surgery	[always/ sometimes/ never]	
	1.21.4 Prenatal and pregnancy care	[always/ sometimes/ never]	
	1.21.5 Children surveillance and preventive care	[always/ sometimes/ never]	
	1.21.6 Adults screening and preventive programs	[always/ sometimes/ never]	
	1.21.7 Assessment/medical certification for social services/social insurance purposes	[always/ sometimes/ never]	
**1.22**	What are the forms of services provided by family physician:		

	1.22.1	Office consultation	[yes/no]	
	1.22.2	Home visits	[yes/no]	
	1.22.3	Telephone consultations	[yes/no]	
	1.22.4	Group sessions	[yes/no]	
	1.22.5.	Assessment for social services	[yes/no]	
	1.22.6	Other	[*please name it*]	
**1.23**	What are the forms of patients records		[EMR only/ both paper and EMR/ paper only]	
**1.24**	What is the level of computerization of family practices in the country:			
	1.24.1	For administrative purposes	[nearly all practices computerized/ more than half/ some/ none]	
	1.24.2	For clinical care (EMR)	[nearly all practices computerized/ more than half/ some/ none]	
	1.24.3	Other reasons	[nearly all practices computerized/ more than half/ some/ none]	
**1.25**	Rank the following organizational forms of family practices:		1. [predominant]<br>2. [less frequent]<br>3. [rare]<br>4. [not existing]	
	1.25.1	Single handed (solo)		
	1.25.2	Group practice of 2 or 3 family physicians		
	1.25.3	Health centers with many family physicians		
	1.25.4	Health centers with family physicians and other specialists		
**1.26**	Total nr. of specialists in family medicine and total number		[n1][n2]	

	of all physicians in the country.			
**1.27**	Average age of working family doctors in the country		[*n*]	
**1.28**	Is there a shortage of family physicians in the country?		[severe/moderate/not at all]	
**1.29**	What is yearly number of qualified GPs/FDs emigrating/immigrating		[*n*] (*please enter the year*) [not available]	
**1.30**	Is WONCA definition of family medicine recognized and implemented by health authorities and policy makers?		[yes/no]	
**Area II: Quality of care**				
**2.1**	Please name important voluntary mechanisms in your country to maintain and improve the quality of care (e.g. clinical guidelines, voluntary peer-review mechanisms).		[voluntary quality improvement mechanism]	
**2.2**	Have evidence-based clinical guidelines been produced for specific use by family physicians?		[yes/no]	
**2.3**	Is there any system of registration and reporting medical errors in PHC in function?		[yes/no]	
**2.4**	2.41	Is there an accreditation system of family practices in the country?	[obligatory/ voluntary/ do not exist]	
	2.42	If one exists, who is responsible for it?	[governmental body/ GP association/ other NGO/other (please, name it)]	
**2.5**	Which part of family practices is accredited?		[nearly all/ majority/ minority/ very few]	
**2.6**	Are there any incentives to undergo accreditation process?		[financial (e.g. higher fee)/better chance to get the contract/internal motivation only/ other (please describe briefly)]	
**2.7**	2.7.1	Do family practices undergo any other common accreditation (e.g. ISO)?	[nearly all/majority/minority/very few/any]	
	2.7.2	If they undergo – please name the most popular accreditation systems	[....]	
**2.8**	2.8.1	Do peer-review groups exist in your country?	[yes/no]	
	2.8.2	If [yes], what part of family physicians is involved?	[nearly all/ majority/ minority/ very few]	
**2.9**	Are there any incentives to participate in the peer-review process?		[financial *(e.g. higher fee)/*better chance to get the contract/internal motivation only/ other *(please*	

		describe briefly)]	
**2.10**	Is your country represented in EQUiP?	[yes/no]	
**Area III: Teaching**			
**3.1**	How many medical faculties at the university level are there in your country? (some of universities may have more than one faculty)	[*n*]	
**3.2**	How many medical faculties have departments of family medicine?	[*n*]	
**3.3**	How many departments are chaired by family physicians with professor title?	[*n*]	
**3.4**	How many departments are chaired by professors of other disciplines?	[*n*]	
**3.5**	% of medical faculties at university level with a postgraduate program in family medicine	[*n*]	
**3.6**	3.61 Is family medicine subject in the undergraduate medical curriculum?	[yes/no]	
	3.62 If [yes], how many universities teaches it?		
**3.7**	3.71 Is there within medical undergraduate curriculum minimum number of teaching hours in family medicine?	[yes/no]	
	3.72 If [yes] – how many?	[*n*]	
**3.8**	Are there any quality measures or requirements for teaching practices?	[none/ yes, but not applied /yes, applied / accreditation necessary]	
**3.9**	Who organize specialist training in family medicine?	[special postgraduate centre/ universities/ hospitals/ GP association/ other/ not applicable – there is no specialty in family medicine/	
**3.10**	What is the duration of specialist training in family medicine in your country (in months)?	[*n*]	
**3.11**	What is the duration of specialist training in family medicine in primary care setting (in months)?	[*n*]	
**3.12**	Are the residents (trainees) in family practice paid?	[not paid/paid by trainers/paid by state or local	

			government/paid by insurance/paid by hospital]	
**3.13**	3.13.1	Is there any formal exam at the end of specialist training?	[yes/no]	
	3.13.2	If [yes]	[MCQ/ written/ oral/ practical/ other (please, describe briefly)]	
**3.14**	3.14.1	Are there in your country any courses for family medicine teachers available (e.g. Leonardo EURACT)?	[yes/no]	
	3.14.2	If [yes], how many family medicine teachers completed them?	[*n*]	
**3.15**	What are the incentives to undertake specialty training in family medicine?		[better professional perspectives/ higher salary as a trainee/ higher future salary/ easy access to specialization/ other *(please describe briefly)*/ no special incentives]	
**3.16**	What is the number of trainees starting (yearly) the postgraduate program in GP ?		[*n*]	
**3.17**	Is there any formal recertification of family physicians in your country?		[voluntary/ obligatory/ not at all]	
**3.18**	Is your country represented in EURACT?		[yes/no]	
**3.19**	Has EURACT Educational Agenda been translated and/or implemented in the country?		[translated and implemented/translated but not implemented/ not translated but implemented/ not translated and not implemented]	
**Area IV: Research**				
**4.1**	Are there postgraduate programs (Ph.D.) in GP/FM available and functioning		[no/ yes, less than 5GPs in program/yes, more than 5GPs in the program]	
**4.2**	Do scientific organization of family physicians exist in your country?		[yes/no]	
**4.3**	Is this organization full member of WONCA?		[yes/no]	
**4.4**	Is your country represented in EGPRN?		[yes/no]	
**4.5**	4.5.1	Is there a scientific peer-reviewed journal on family medicine published in this country?	[yes/no]	

	4.5.2	If [yes], how many issues a year are published?	[*n*]	
**4.6**	4.6.1	Are there scientific conferences on national level regularly being organized?	[yes/no]	
	4.6.2	If [yes], how often?	[twice a year or more often/annually/biannually/other *(please describe briefly)*]	
**4.7**	How many GPs do usually attend WONCA Europe conference		[less than 5/ 5-15/15-30/more than 30]	
**4.8**	How many abstracts are presented at WONCA conferences		[n]	
**4.9**	Are there special funds to carry on research in family medicine available?		[yes/no]	
**4.10**	4.10.1	Is there in the country a research network of family physicians/family practices?	[yes/no]	
	4.10.1	If [yes] how many doctors/practices are involved?	[*n*]	
